# Association between vitamin D deficiency and incident varicose veins: a propensity score-matched cohort study

**DOI:** 10.3389/fnut.2026.1870207

**Published:** 2026-06-25

**Authors:** Kuo-Mao Lan, Wei-Ting Wang, Ying-Jen Chang

**Affiliations:** 1 Department of Anesthesiology, Chi Mei Hospital, Liouying, Tainan City, Taiwan; 2 Department of Anesthesiology, E-Da Hospital, I-Shou University, Kaohsiung City, Taiwan; 3 Department of Anesthesiology, Chi Mei Medical Center, Tainan City, Taiwan; 4 Department of Recreation and Health-Care Management, College of Recreation and Health Management, Chia Nan University of Pharmacy and Science, Tainan City, Taiwan

**Keywords:** chronic venous disease, cohort study, propensity score matching, TriNetX, varicose veins, vitamin D deficiency

## Abstract

**Background:**

Evidence linking vitamin D deficiency (VDD) to varicose veins remains limited and largely derived from small or cross-sectional studies. We examined whether VDD is associated with incident varicose veins in a large real-world cohort.

**Methods:**

Using the TriNetX Global Collaborative Network, we conducted a retrospective propensity score–matched cohort study of adults aged ≥40 years with at least two serum 25-hydroxyvitamin D [25(OH)D] measurements between 2010 and 2023. VDD was defined as two measurements ≤19.9 ng/mL, and controls had two measurements ≥30.0 ng/mL. A 1-year landmark design was applied. The primary outcome was 5-year incident varicose veins of the lower extremities. Secondary outcomes included varicose veins with ulceration, inflammation, and other complications. Cox proportional hazards models were used to estimate hazard ratios (HRs) with 95% confidence intervals (CIs).

**Results:**

After matching, 257,651 patients were included in each cohort. VDD was associated with a higher risk of incident varicose veins (HR 1.62, 95% CI 1.50–1.75; *p* < 0.001). Increased risks were also observed for varicose veins with ulceration (HR 2.22), inflammation (HR 2.71), and complications (HR 1.45) (all *p* < 0.001). Findings were consistent across sensitivity and subgroup analyses. Vitamin D insufficiency (20.0–29.9 ng/mL) was also associated with increased risk (HR 1.56, *p* < 0.001).

**Conclusion:**

VDD was associated with an increased risk of incident varicose veins in this large cohort. This association was also observed among older adults and individuals with diabetes, suggesting potential effect modification by age and diabetic status. These findings suggest that reduced vitamin D status may be relevant to venous disease risk, although causality cannot be inferred. Further prospective studies are needed to determine whether correction of VDD can modify this risk.

## 1 Introduction

Varicose veins of the lower extremities are among the most common manifestations of chronic venous disease and affect approximately 23% of adults in the United States (1, 2). Although often perceived as a cosmetic condition, varicose veins are associated with leg symptoms, impaired quality of life, and progression to more advanced venous disease, including skin changes, superficial thrombophlebitis, and venous ulceration (3–5). Established risk factors include older age, female sex, pregnancy, obesity, prolonged standing, and genetic susceptibility (6–8). Despite the recognition of these risk factors, the potential contribution of nutritional and metabolic factors to varicose vein development remains incompletely understood.

Vitamin D deficiency (VDD), commonly defined as a serum 25-hydroxyvitamin D [25(OH)D] concentration below 20 ng/mL, is highly prevalent worldwide and has been estimated to affect more than one billion people (9, 10). Beyond its classical role in calcium homeostasis and skeletal health, vitamin D has been implicated in vascular biology through effects on endothelial function, oxidative stress, inflammatory signaling, and regulation of the renin-angiotensin system. These mechanisms are biologically relevant to venous wall remodeling and chronic venous disease. In addition, recent ex vivo evidence has suggested that active vitamin D may attenuate oxidative stress and modulate nitric oxide synthase-related pathways in varicose venous tissue (11), providing a plausible mechanistic rationale for a link between vitamin D status and venous disease.

However, clinical epidemiological evidence on the association between VDD and incident varicose veins remains limited. Existing studies appear to be predominantly small-scale, cross-sectional, or mechanistic in nature, and a Mendelian randomization study evaluating genetically predicted circulating 25-hydroxyvitamin D did not support a clear association with varicose vein risk (12). Accordingly, whether clinically defined VDD is associated with the subsequent development of varicose veins in real-world populations remains uncertain. To address this gap, we conducted a propensity score-matched cohort study using the TriNetX Global Collaborative Network to examine the association between VDD and the 5-year risk of incident varicose veins of the lower extremities, and to further explore whether a gradient pattern exists across vitamin D status categories.

## 2 Methods

### 2.1 Data sources and exposure definition

This retrospective cohort study was conducted using electronic health record data from the TriNetX Global Collaborative Network, a federated real-world data platform comprising 171 healthcare organizations across multiple countries (13). The TriNetX database has been widely used in real-world clinical research across multiple medical specialties (14–16). The study was approved by the Institutional Review Board of Chi Mei Medical Center, with a waiver of informed consent, and was conducted in accordance with the Declaration of Helsinki. Because all data were de-identified and HIPAA-compliant, individual patient consent was not required.

Eligible participants were adults aged ≥40 years who had at least two serum 25-hydroxyvitamin D [25(OH)D] measurements on record between January 1, 2010, and December 31, 2023. Two mutually exclusive cohorts were formed based on 25(OH)D status. The VDD cohort included individuals with two distinct serum 25-hydroxyvitamin D [25(OH)D] measurements of ≤19.9 ng/mL, with the second qualifying value designated as the index date. To confirm exposure stability, patients in the VDD group were excluded if any 25(OH)D value ≥30.0 ng/mL had been recorded in the 3 years preceding the index date. The reference cohort comprised patients with two separate 25(OH)D measurements of ≥30.0 ng/mL, with the index date assigned in the same manner. Individuals in the reference cohort were excluded if any 25(OH)D value of ≤19.9 ng/mL had been documented during the same three-year look-back period.

### 2.2 Exclusion criteria

Patients with a pre-existing diagnosis of varicose veins of the lower extremities were excluded to ensure incident-only ascertainment. A one-year landmark period was further applied, during which newly coded varicose vein diagnoses served as an additional exclusion criterion to reduce reverse causation. A 1-year landmark period was selected, given the chronic and slowly progressive nature of varicose vein development. Patients were also excluded if they had prior diagnoses of conditions that could independently influence vitamin D metabolism, venous hemodynamics, or venous disease risk, including advanced renal disease, prior bariatric surgery, severe hepatic dysfunction, inflammatory bowel disease, malabsorptive disorders, and selected cardiovascular or venous comorbidities. Complete code-level definitions for all exclusion criteria are provided in Supplementary Table 1.

### 2.3 Data collection and propensity score matching

Baseline characteristics were extracted across four domains: demographics, clinically relevant comorbidities, medication use, and laboratory indices. Variables were selected based on their known or biologically plausible role as confounders of the exposure–outcome relationship. Of particular relevance to varicose vein pathogenesis, the matching model explicitly incorporated obesity, pregnancy history, use of exogenous estrogens and systemic contraceptives, nicotine dependence, and anticoagulant use; variables known to affect vitamin D metabolism or status—including glucocorticoid use and vitamin D supplementation—were additionally included to ensure comparability of the underlying nutritional milieu. A full list of covariates is provided in Supplementary Table 1. One-to-one propensity score matching (PSM) was performed using a greedy nearest-neighbor algorithm with a caliper of 0.1 standard deviations of the logit-transformed propensity score. Covariate balance was assessed using standardized mean differences (SMDs), with values <0.10 indicating adequate balance.

### 2.4 Primary and secondary outcomes

The primary outcome was incident varicose veins of the lower extremities (ICD-10-CM I83), ascertained over the subsequent 4-year follow-up period (days 365–1825), within a total 5-year observation window from the index date. Secondary outcomes included clinically meaningful subtypes: varicose veins with ulceration, varicose veins with inflammation, and varicose veins with other complications. Detailed code-based definitions for these outcomes are provided in Supplementary Table 1.

### 2.5 Validation of study design

Two prespecified control outcomes were used to assess the validity of the study design. Osteoporotic fracture served as a positive control because vitamin D has a well-established role in calcium homeostasis and bone mineralization; therefore, an increased risk in the VDD cohort would support the internal validity of the analysis. Appendicitis was selected as a negative control because its pathogenesis is not biologically linked to vitamin D status; accordingly, a null association would reduce concern about substantial unmeasured confounding or systematic detection bias. In addition, we compared the overall rate of healthcare encounters between the matched cohorts to evaluate whether they had similar levels of medical contact, and thus comparable opportunities for outcome detection, during follow-up.

### 2.6 Sensitivity and subgroup analyses

Three prespecified sensitivity analyses were performed to examine the robustness of the primary findings against potential bias. Model I limited the study period to 2016–2023 to ensure coding consistency after full adoption of ICD-10-CM. Model II excluded patients who died during follow-up as a sensitivity analysis to assess whether competing mortality materially influenced the observed association. Model III included only patients with at least one ambulatory encounter during follow-up to address possible underascertainment of outcomes among clinically inactive individuals. Prespecified subgroup analyses were also conducted according to sex, age (40–65 vs. >65 years), obesity, hyperlipidemia, history of cancer, diabetes mellitus, and hypertension. Within each subgroup, propensity score matching and time-to-event analyses were repeated using the same analytic framework as in the primary analysis, and interaction testing was performed to assess potential effect modification.

### 2.7 Additional analysis

To examine whether a less severe reduction in vitamin D status was likewise associated with varicose vein risk, and to assess the presence of a possible exposure–response pattern, we performed an additional analysis using a vitamin D insufficiency (VDI) cohort. VDI was defined as two separate serum 25-hydroxyvitamin D [25(OH)D] measurements of 20.0–29.9 ng/mL. This cohort was compared with the same vitamin D–sufficient reference group, defined by serum 25(OH)D levels of at least 30.0 ng/mL. To preserve comparability with the primary analysis, the same eligibility criteria, exclusion criteria, propensity score matching variables, and statistical methods were applied.

### 2.8 Statistical analysis

Time-to-event relationships were examined using Cox proportional hazards regression models, and the results are presented as hazard ratios (HRs) with corresponding 95% confidence intervals (CIs). The proportional hazards assumption for each model was evaluated using Schoenfeld residuals. Kaplan–Meier analysis was used to illustrate event-free survival over time, and differences between groups were assessed with the log-rank test. In addition to the primary propensity score–matched analysis, a multivariable Cox regression model was performed in the unmatched cohort to examine the independent association between VDD and outcomes. For the primary outcome, E-values were additionally calculated to estimate the minimum strength of an unmeasured confounder that would be necessary to fully explain the observed association. Statistical significance for the primary analysis was defined as a two-sided *p* value <0.05. In contrast, analyses of secondary outcomes, sensitivity analyses, subgroup analyses, and design-validation outcomes were considered exploratory in nature and were therefore interpreted without adjustment for multiple comparisons. All statistical analyses were performed directly within the TriNetX platform, and missing data were handled as recorded in the source database, without imputation. Competing risk analysis was not performed due to platform limitations of TriNetX.

## 3 Results

### 3.1 Baseline characteristics

Following the application of eligibility and exclusion criteria, 291,819 patients with VDD and 999,076 individuals with sufficient vitamin D levels were identified (Supplementary Figure 1). After 1:1 propensity score matching, 257,651 patients were retained in each cohort. Prior to matching, the two groups differed substantially across multiple covariates, most notably age, race, obesity, and medication use. After matching, covariate balance was achieved across all variables, with all SMDs falling below 0.10 (Table 1). The median follow-up duration was 1,825 days in both cohorts, with mean follow-up of 1,573 days and 1,583 days in the VDD and control groups, respectively.

**Table 1:** Baseline characteristics of patients with vitamin D deficiency and sufficient vitamin D levels.

Variables	Before matching			After matching		
	VDD group (*n* = 291,819)	Control group (*n* = 999,076)	SMD	VDD group (*n* = 257,651)	Control group (*n* = 257,651)	SMD
Patient characteristics						
Age at index (years)	53.5 ± 14.1	60.8 ± 13.3	0.535	54.8 ± 14.1	55.2 ± 13.5	0.025
BMI ≥ 30 (kg/m^2^)	88,828 (30.4)	221,276 (22.1)	0.189	71,448 (27.7)	74,372 (28.9)	0.025
Female	197,915 (67.8)	740,104 (74.1)	0.138	177,282 (68.8)	175,000 (67.9)	0.019
White	133,258 (45.7)	762,684 (76.3)	0.663	131,917 (51.2)	130,144 (50.5)	0.014
Black or African American	68,619 (23.5)	75,483 (7.6)	0.452	47,038 (18.3)	47,616 (18.5)	0.006
Asian	13,039 (4.5)	42,175 (4.2)	0.012	12,645 (4.9)	11,954 (4.6)	0.013
Comorbidities and healthcare utilization						
Essential (primary) hypertension	84,602 (29.0)	332,838 (33.3)	0.093	73,077 (28.4)	75,064 (29.1)	0.017
Hyperlipidemia	72,866 (25.0)	369,025 (36.9)	0.261	66,595 (25.8)	68,300 (26.5)	0.015
Encounter for general examination	59,626 (20.4)	278,385 (27.9)	0.174	53,587 (20.8)	53,394 (20.7)	0.002
Neoplasms	51,994 (17.8)	233,505 (23.4)	0.138	46,976 (18.2)	46,508 (18.1)	0.005
Other nutritional deficiencies	48,161 (16.5)	272,016 (27.2)	0.262	45,501 (17.7)	47,700 (18.5)	0.022
Overweight and obesity	48,813 (16.7)	109,354 (10.9)	0.168	37,923 (14.7)	38,945 (15.1)	0.011
Diabetes mellitus	40,958 (14.0)	119,645 (12.0)	0.061	33,924 (13.2)	34,811 (13.5)	0.010
Gastro-esophageal reflux disease	37,706 (12.9)	162,654 (16.3)	0.095	33,197 (12.9)	33,894 (13.2)	0.008
Disorders of thyroid gland	33,577 (11.5)	187,235 (18.7)	0.203	31,299 (12.1)	31,431 (12.2)	0.002
Nicotine dependence	26,946 (9.2)	46,522 (4.7)	0.181	19,355 (7.5)	19,470 (7.6)	0.002
Ischemic heart diseases	13,196 (4.5)	57,294 (5.7)	0.055	11,780 (4.6)	12,057 (4.7)	0.005
Diseases of liver	12,043 (4.1)	37,199 (3.7)	0.021	10,181 (4.0)	10,097 (3.9)	0.002
Iron deficiency anemia	12,479 (4.3)	30,781 (3.1)	0.064	9,619 (3.7)	9,777 (3.8)	0.003
Chronic kidney disease (CKD)	9,647 (3.3)	45,440 (4.5)	0.064	8,673 (3.4)	8,940 (3.5)	0.006
Cerebrovascular diseases	8,962 (3.1)	35,855 (3.6)	0.029	7,731 (3.0)	8,007 (3.1)	0.006
COPD	8,684 (3.0)	27,792 (2.8)	0.012	7,391 (2.9)	7,295 (2.8)	0.002
Pregnancy, childbirth and the puerperium	8,188 (2.8)	12,926 (1.3)	0.107	5,729 (2.2)	5,527 (2.1)	0.005
Alcohol related disorders	6,545 (2.2)	11,286 (1.1)	0.087	4,569 (1.8)	4,480 (1.7)	0.003
Systemic connective tissue disorders	4,587 (1.6)	21,368 (2.1)	0.042	4,196 (1.6)	4,206 (1.6)	0.000
COVID-19	3,798 (1.3)	12,894 (1.3)	0.001	3,293 (1.3)	3,159 (1.2)	0.005
Malnutrition	2,442 (0.8)	5,130 (0.5)	0.039	1887 (0.7)	1898 (0.7)	0.000
Laboratory data						
Hemoglobin ≥ 12 g/dL	175,956 (60.3)	570,513 (57.1)	0.065	153,365 (59.5)	154,975 (60.1)	0.013
Albumin ≥3.5 g/dL	154,980 (53.1)	530,652 (53.1)	0.000	134,627 (52.3)	136,699 (53.1)	0.016
HbA1c ≥ 9%	14,900 (5.1)	19,051 (1.9)	0.175	10,261 (4.0)	10,658 (4.1)	0.008
eGFR ≥ 60 mL/min/1.73 m^2^	158,902 (54.5)	536,989 (53.7)	0.014	136,283 (52.9)	135,598 (52.6)	0.005
Medications						
Cardiovascular system	133,700 (45.8)	500,274 (50.1)	0.085	114,917 (44.6)	116,036 (45.0)	0.009
Glucocorticoids	72,782 (24.9)	285,490 (28.6)	0.082	63,351 (24.6)	63,906 (24.8)	0.005
Vitamin d supplementation	27,953 (9.6)	172,356 (17.3)	0.227	26,455 (10.3)	27,681 (10.7)	0.016
Anticoagulants	30,375 (10.4)	89,069 (8.9)	0.051	24,822 (9.6)	25,337 (9.8)	0.007
Platelet aggregation inhibitors	28,439 (9.7)	117,345 (11.7)	0.065	24,520 (9.5)	24,952 (9.7)	0.006
Insulins and analogues	20,345 (7.0)	41,916 (4.2)	0.121	15,582 (6.0)	15,937 (6.2)	0.006
Contraceptives, systemic	9,852 (3.4)	26,574 (2.7)	0.042	8,032 (3.1)	7,698 (3.0)	0.008
Estrogens	5,424 (1.9)	47,989 (4.8)	0.165	5,247 (2.0)	5,311 (2.1)	0.002

Data are presented as *n* (%) for categorical variables and mean ± standard deviation for continuous variables. Standardized mean differences (SMDs) were used to assess covariate balance between groups, with an absolute SMD <0.10 indicating adequate balance. BMI ≥30 kg/m^2^ was defined by measured body mass index, whereas “overweight and obesity” was defined by ICD-10-CM diagnostic codes. BMI, body mass index; CKD, chronic kidney disease; COPD, chronic obstructive pulmonary disease; COVID-19, coronavirus disease 2019; eGFR, estimated glomerular filtration rate; HbA1c, hemoglobin A1c; SMD, standardized mean difference; VDD, vitamin D deficiency.

### 3.2 Outcomes

**Figure 1:**
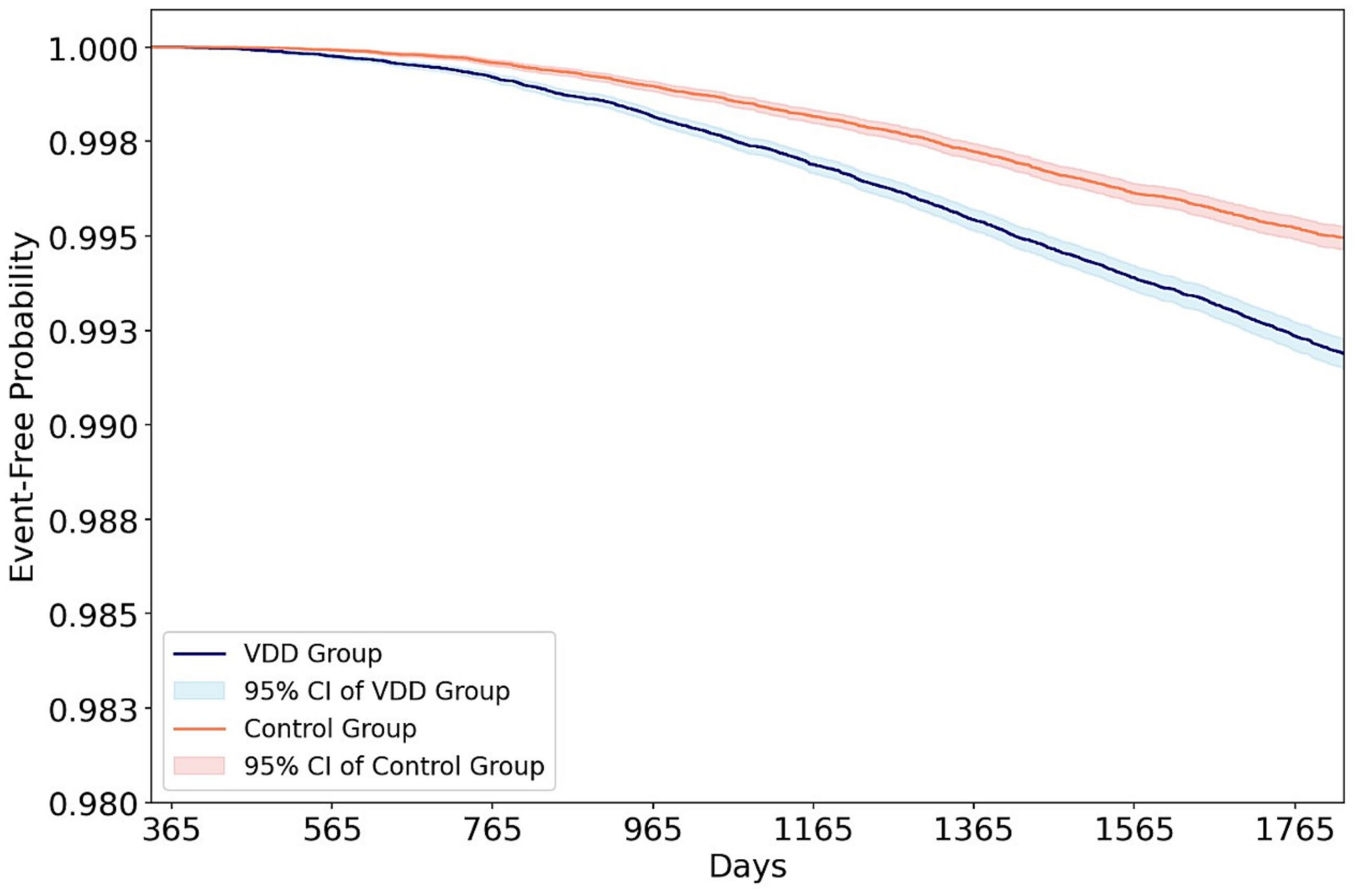
Kaplan–Meier curves for event-free probability of incident varicose vein over 5 years of follow-up in the propensity score-matched cohorts. The curves compare patients with vitamin D deficiency and matched controls with sufficient vitamin D status. A 1-year landmark period was applied, and between-group differences were assessed using the log-rank test. VDD, vitamin D deficiency.

Over the 4-year observation window following the 1-year landmark period, incident varicose veins of the lower extremities were recorded in 1,665 patients (0.65%) in the VDD group and 1,031 patients (0.40%) in the control group. VDD was associated with a significantly higher risk of incident varicose veins (HR 1.62, 95% CI 1.50–1.75; *p* < 0.001) (Table 2). The E-value for the point estimate was 2.62, and for the lower confidence interval boundary was 2.37. Kaplan–Meier analysis demonstrated a consistently lower event-free probability in the VDD cohort over time (log-rank *p* < 0.001) (Figure 1). With respect to secondary outcomes, VDD was similarly associated with varicose veins with ulceration (HR 2.22, *p* < 0.001), varicose veins with inflammation (HR 2.71, *p* < 0.001), and varicose veins with other complications (HR 1.45, *p* < 0.001).

**Table 2:** Association between vitamin D deficiency and the 5-year risk of incident varicose vein.

Outcome	VDD group (*n* = 257,651)	Control group (*n* = 257,651)	HR (95% CI)	*p* value
	Events (%)	Events (%)		
Primary outcome				
Varicose vein of LE	1,665 (0.65%)	1,031 (0.40%)	1.62 (1.50–1.75)	<0.001
Secondary outcomes				
Varicose vein with ulcer	171 (0.07%)	77 (0.03%)	2.22 (1.70–2.91)	<0.001
Varicose vein with inflammation	276 (0.11%)	102 (0.04%)	2.71 (2.16–3.40)	<0.001
Varicose vein with complications	687 (0.27%)	473 (0.18%)	1.45 (1.29–1.63)	<0.001

CI, confidence interval; HR, hazard ratio; VDD, vitamin D deficiency; LE, lower extremities; Outcomes were assessed from 1 year after index date (days 365–1825).

### 3.3 Validation of study design and healthcare utilization

The positive control outcome, osteoporotic fracture, was more frequently observed in the VDD group (HR 1.45, 95% CI 1.28–1.64; *p* < 0.001), consistent with the known biological relationship between vitamin D and bone health. The negative control outcome, appendicitis, showed no significant difference between groups (HR 0.97, 95% CI 0.78–1.19; *p* = 0.734). Healthcare encounter rates were high in both cohorts (95.20% vs. 97.11%), with a small but statistically significant difference (HR 0.90; *p* < 0.001), supporting broadly comparable opportunity for outcome ascertainment across groups (Table 3).

**Table 3:** Validation of study design and healthcare utilization.

Outcome	VDD group (*n* = 257,651)	Control group (*n* = 257,651)	HR (95% CI)	*p* value
	Events (%)	Events (%)		
Positive control outcome				
Osteoporotic fracture	607 (0.24%)	419 (0.16%)	1.45 (1.28–1.64)	<0.001
Negative control outcome				
Appendicitis	175 (0.07%)	182 (0.07%)	0.97 (0.78–1.19)	0.734
Healthcare utilization validation				
Healthcare visit	245,295 (95.20%)	250,210 (97.11%)	0.90 (0.90–0.91)	<0.001

Data are presented as the number of events (percentage) and hazard ratios (HRs) with 95% confidence intervals (CIs). CI, confidence interval; HR, hazard ratio; VDD, vitamin D deficiency; Outcomes were assessed from 1 year after index date (days 365–1825).

### 3.4 Sensitivity analyses and subgroup analyses

The association between VDD and incident varicose veins was consistent across all three prespecified sensitivity analyses (Table 4). When the study period was restricted to 2016–2023 to ensure ICD-10-CM coding consistency (Model I), the association remained significant (HR 1.45, *p* < 0.001). Restriction to patients who survived throughout follow-up (Model II) yielded a comparable estimate (HR 1.53, *p* < 0.001), as did restriction to patients with at least one ambulatory encounter during follow-up (Model III; HR 1.59, *p* < 0.001). Findings for all secondary outcomes were likewise consistent across sensitivity models.

**Table 4:** Sensitivity analyses of the association between vitamin D deficiency and the 5-year risk of incident varicose vein.

Outcomes	Model I		Model II		Model III	
	HR (95% CI)	*p* value	HR (95% CI)	*p* value	HR (95% CI)	*p* value
Varicose vein of LE	1.45 (1.34–1.58)	<0.001	1.53 (1.42–1.66)	<0.001	1.59 (1.47–1.71)	<0.001
Varicose vein with ulcer	2.03 (1.53–2.70)	<0.001	2.16 (1.62–2.87)	<0.001	2.04 (1.57–2.65)	<0.001
Varicose vein with inflammation	2.37 (1.79–3.14)	<0.001	2.39 (1.90–2.99)	<0.001	2.78 (2.22–3.48)	<0.001
Varicose vein with complications	1.36 (1.20–1.53)	<0.001	1.41 (1.25–1.58)	<0.001	1.46 (1.30–1.64)	<0.001

Model I restricted the study period to 2016–2023 to ensure coding consistency. Model II excluded patients who died during follow-up. Model III was restricted to patients with at least one ambulatory healthcare encounter during follow-up to address potential differences in outcome ascertainment. CI, confidence interval; HR, hazard ratio; LE, lower extremities.

Subgroup analyses revealed that the association between VDD and varicose vein risk was present across all prespecified subgroups (Table 5). Statistically significant effect modification was identified for age (*p* for interaction = 0.005), with a stronger association observed in patients older than 65 years (HR 1.77) than in those aged 40–65 years (HR 1.41). Significant interactions were also detected for obesity status (*p* = 0.048) and diabetes mellitus (*p* = 0.039), with higher HRs observed in the non-obese (HR 1.61) and diabetic (HR 1.80) subgroups, respectively. No significant interactions were observed for sex, hyperlipidemia, cancer history, or hypertension.

**Table 5:** Subgroup analysis.

Subgroup	HR (95% CI)	*p* value	*p* for interaction
Male	1.62 (1.37–1.90)	<0.001	Reference
Female	1.58 (1.45–1.73)	<0.001	0.794
40–65 years	1.41 (1.27–1.58)	<0.001	Reference
>65 years	1.77 (1.59–1.98)	<0.001	0.005
Obesity	1.37 (1.20–1.56)	<0.001	Reference
No obesity	1.61 (1.47–1.78)	<0.001	0.048
Hyperlipidemia	1.64 (1.47–1.83)	<0.001	Reference
No hyperlipidemia	1.45 (1.30–1.61)	<0.001	0.117
Cancer history	1.69 (1.48–1.93)	<0.001	Reference
No cancer history	1.43 (1.31–1.57)	<0.001	0.05
DM	1.80 (1.52–2.13)	<0.001	Reference
No DM	1.45 (1.33–1.59)	<0.001	0.039
HTN	1.65 (1.48–1.84)	<0.001	Reference
No HTN	1.44 (1.30–1.61)	<0.001	0.083

Prespecified subgroup analyses were also conducted according to sex, age (40–65 vs. >65 years), obesity, hyperlipidemia, history of cancer, diabetes mellitus, and hypertension.

### 3.5 Multivariable Cox regression analysis

In the multivariable Cox proportional hazards model, VDD remained independently associated with incident varicose veins after adjustment for all covariates (HR 1.66, 95% CI 1.56–1.76; *p* < 0.001) (Table 6). Among the remaining covariates, overweight/obesity (HR 1.56), White race (HR 1.54), and male sex (HR 0.70, protective) demonstrated the strongest independent associations with varicose vein risk. Modest but statistically significant associations were additionally observed for GERD (HR 1.20), liver disease (HR 1.17), iron deficiency anemia (HR 1.13), thyroid disorders (HR 1.12), hypertension (HR 1.11), neoplasms (HR 1.09), and older age (HR 1.02 per year).

**Table 6:** Multivariable Cox proportional hazards regression analysis for incident varicose veins of the lower extremities.

Variable	HR	(95% CI)	*p*-value
VDD vs. Control groups	1.66	(1.56–1.76)	<0.001
Male vs. Female	0.70	(0.66–0.75)	<0.001
Age at index	1.02	(1.02–1.02)	<0.001
White	1.54	(1.45–1.63)	<0.001
Essential hypertension	1.11	(1.04–1.17)	0.001
Hyperlipidemia	1.01	(0.95–1.07)	0.836
Neoplasms	1.09	(1.03–1.15)	0.003
Overweight / Obesity	1.56	(1.46–1.67)	<0.001
Diabetes mellitus	1.06	(0.99–1.14)	0.110
GERD	1.20	(1.13–1.27)	<0.001
Thyroid disorders	1.12	(1.06–1.19)	<0.001
Nicotine dependence	0.95	(0.86–1.05)	0.323
Liver disease	1.17	(1.05–1.30)	0.005
Iron deficiency anemia	1.13	(1.00–1.27)	0.043
CKD	1.07	(0.96–1.19)	0.241
Alcohol-related disorders	0.95	(0.77–1.18)	0.663

HR, hazard ratio; CI, confidence interval; CKD, chronic kidney disease; VDD: vitamin D deficiency; Age was modeled as a continuous variable (per 1-year increase).

### 3.6 Vitamin D insufficiency and risk of varicose vein

Among 317,566 matched pairs in the vitamin D insufficiency (VDI) analysis, VDI was likewise associated with a higher risk of incident varicose veins of the lower extremities (HR 1.56, 95% CI 1.47–1.67; *p* < 0.001). Associations were also observed for varicose veins with ulceration (HR 1.55, *p* < 0.001), inflammation (HR 1.79, *p* < 0.001), and other complications (HR 1.52, *p* < 0.001), suggesting a gradient pattern across vitamin D status categories (Table 7).

**Table 7:** Association between vitamin D insufficiency (20.0–29.9 ng/mL) and the 5-year risk of incident varicose vein.

Outcome	VDI group (*n* = 317,566)	Control group (*n* = 317,566)	HR (95% CI)	*p* value
	Events (%)	Events (%)		
Varicose vein of LE	2,423 (0.76%)	1,541 (0.49%)	1.56 (1.47–1.67)	<0.001
Varicose vein with ulcer	195 (0.06%)	125 (0.04%)	1.55 (1.24–1.94)	<0.001
Varicose vein with inflammation	289 (0.09%)	160 (0.05%)	1.79 (1.48–2.17)	<0.001
Varicose vein with complications	1,057 (0.33%)	690 (0.22%)	1.52 (1.38–1.67)	<0.001

CI, confidence interval; HR, hazard ratio; VDI, vitamin D insufficiency; LE, lower extremities; Outcomes were assessed from 1 year after index date (days 365–1825).

## 4 Discussion

Despite the high global prevalence of varicose veins and the growing recognition of vitamin D’s vascular roles, large-scale longitudinal data examining the relationship between VDD and the incident risk of varicose veins have remained absent. Prior evidence has been confined to small mechanistic investigations or cross-sectional observations, leaving the epidemiological question unresolved. In the present propensity score-matched cohort of more than 515,000 matched individuals, VDD was associated with a significantly elevated five-year risk of incident varicose veins of the lower extremities (HR 1.62, 95% CI 1.50–1.75), with consistent associations observed across all secondary clinical subtypes, three prespecified sensitivity models, and a fully adjusted multivariable Cox regression model. These findings represent, to our knowledge, one of the largest real-world epidemiological analyses to date characterizing the association between VDD and varicose vein risk.

The present findings align with the broader body of evidence linking low vitamin D status to venous and vascular disease. Prior observational studies and meta-analyses have documented an association between VDD and venous thromboembolism, suggesting that low vitamin D status may be broadly relevant to venous vascular health (17, 18), though the underlying mechanisms likely differ across disease entities. At the tissue level, a recent *ex vivo* pilot study demonstrated that the active form of vitamin D attenuates reactive oxygen species generation and modulates nitric oxide synthase isoform expression in varicose venous samples from both obese and non-obese patients (11), providing direct molecular evidence of vitamin D’s involvement in venous wall biology.

A prior Mendelian randomization study did not demonstrate a significant association between genetically predicted vitamin D levels and varicose vein risk. Differences in exposure definition, including lifelong genetically determined levels versus clinically defined deficiency states, may partially account for this discrepancy. Genetic instruments for vitamin D explain only a modest fraction of its circulating variability, and the statistical power of MR analyses for disease endpoints with moderate event rates may be insufficient to detect associations of the magnitude observed here. Furthermore, MR does not capture the episodic or sustained clinical deficiency states that characterize real-world VDD. Taken together, these methodological distinctions suggest that the MR and observational findings address related but distinct scientific questions, and the null MR result does not necessarily negate an epidemiological association between clinically defined VDD and varicose vein risk.

Several biologically plausible mechanisms may explain the observed association between VDD and varicose vein risk. Vitamin D supports endothelial nitric oxide bioavailability and suppresses inducible nitric oxide synthase activity (19–21); therefore, reduced vitamin D status could plausibly contribute to oxidative stress, endothelial dysfunction, and impaired venous wall integrity, although these pathways were not directly examined in the present study. In addition, the vitamin D receptor is expressed in vascular smooth muscle and endothelial cells, where it modulates inflammatory signaling pathways such as NF-κB (22, 23). Reduced vitamin D signaling may be associated with pro-inflammatory cytokine activity, extracellular matrix degradation, venous valve dysfunction, and progressive venous dilation (11, 24); however, these mechanistic links remain inferential in the context of our observational design. Vitamin D also interacts with the renin–angiotensin system, and dysregulation of this pathway may adversely affect venous compliance and pressure (25, 26). Together, these mechanisms provide a biologically plausible explanation for the observed association, but they should be interpreted as hypothesis-generating rather than as evidence of a causal pathway.

An additional consideration is the potential role of magnesium in modifying the association between vitamin D status and varicose vein risk. Magnesium is a required cofactor for key enzymes involved in vitamin D metabolism, including hepatic 25-hydroxylase and renal 1α-hydroxylase, and magnesium deficiency has been shown to impair the conversion of 25-hydroxyvitamin D to its biologically active form, 1,25-dihydroxyvitamin D (calcitriol) (27, 28). Moreover, magnesium independently contributes to vascular smooth muscle regulation, endothelial function, and anti-inflammatory signaling (29–32), all of which are relevant to venous wall homeostasis. Therefore, concurrent magnesium deficiency could theoretically amplify the vascular consequences of VDD or exert independent effects on venous disease risk. As serum magnesium levels were not assessed in the present analysis, the potential confounding or mediating role of magnesium status could not be evaluated, and this represents an important avenue for future investigation.

In current study, although COVID-19 was included as a balanced covariate in the matching model, the low prevalence of COVID-19 in both cohorts (approximately 1.3%) precluded a dedicated subgroup analysis to determine whether the association between VDD and venous disease risk is modified by SARS-CoV-2 infection. Given the well-established hypercoagulable and pro-inflammatory state associated with COVID-19 (33, 34), the potential interaction between VDD and COVID-19 in promoting superficial venous thrombosis and varicose vein complications warrants investigation in future studies with sufficient sample sizes of infected individuals.

A notable finding of the present analysis is that vitamin D insufficiency (VDI; 20.0–29.9 ng/mL) was also associated with an increased risk of incident varicose veins (HR 1.56, 95% CI 1.47–1.67). Similar associations were observed for clinically relevant subtypes, including varicose veins with ulceration, inflammation, and other complications. Although the effect estimate for VDI was numerically lower than that observed for VDD, these analyses were conducted in separate matched cohorts and were not intended for direct comparison. Accordingly, the present findings should not be interpreted as establishing a formal dose–response relationship. Rather, they suggest that the association between reduced vitamin D status and varicose vein risk may extend beyond overt deficiency to include milder degrees of insufficiency. From a clinical perspective, this observation is potentially relevant because it suggests that even moderate reductions in vitamin D status may be associated with venous disease risk, although the underlying mechanisms and causal relevance require further investigation. The higher crude event rate observed in the VDI cohort (0.76%) (Table 7) than in the VDD cohort (0.65%) (Table 2) should be interpreted cautiously. Because the VDI and VDD analyses were performed as separate propensity score–matched comparisons, the matched populations may have differed in baseline characteristics and risk profiles, which could account for this numerical difference rather than indicating a truly higher risk in the VDI group.

Subgroup analyses suggested two clinically interpretable patterns. The association between VDD and varicose vein risk appeared more pronounced in patients older than 65 years (HR 1.77) compared with those aged 40–65 years (HR 1.41; *p* for interaction = 0.005), and in patients with diabetes mellitus (HR 1.80 vs. HR 1.45; *p* for interaction = 0.039). These findings may reflect increased biological vulnerability, as aging and diabetes are both associated with endothelial dysfunction and impaired vascular homeostasis (35–37), which could potentially amplify susceptibility to the vascular effects of VDD. In contrast, the association appeared attenuated in individuals with obesity (HR 1.37 vs. HR 1.61; *p* for interaction = 0.048). This pattern may reflect several non-mutually exclusive mechanisms. Given that obesity is a strong independent risk factor for varicose veins (38–40), the high baseline risk in this subgroup may reduce the relative contribution of VDD on a multiplicative scale. In addition, the known association between obesity and lower circulating 25(OH)D levels may narrow the effective exposure contrast between groups (41, 42), potentially attenuating the observed effect estimate. Consistent with this notion, Damay et al. (43) reported that anthropometric adiposity was associated with impaired endothelial function, further supporting the concept that obesity-related vascular dysfunction may modify vascular susceptibility to additional risk factors such as VDD. Alternatively, residual confounding or differences in healthcare utilization may also contribute. Importantly, the association between VDD and varicose vein risk remained statistically significant across all subgroups.

In the current study, the observed event rates were low (0.65% in the VDD group and 0.40% in the control group over four years) compared with the commonly cited cross-sectional prevalence of varicose veins in the general population, which is approximately 23% (44). This discrepancy is expected because our analysis assessed the incidence of newly diagnosed varicose veins among individuals without pre-existing disease, rather than the overall prevalence of existing varicose veins in the population. In addition, ascertainment based on electronic health records captures only clinically documented diagnoses; therefore, mild or asymptomatic varicose veins may not have been formally coded. Such underascertainment would likely be non-differential between groups and would tend to bias the association toward the null. Thus, the observed association may represent a conservative estimate of the relationship between VDD and incident varicose veins.

The present study benefits from several methodological features that enhance the validity of its findings. The large matched sample provides substantial statistical power, while the one-year landmark period, positive and negative control outcomes, E-value analysis, and three sensitivity models collectively support the robustness and internal validity of the primary association. The E-value of 2.62 for the point estimate indicates that an unmeasured confounder would need to be strongly associated with both VDD and varicose vein risk, beyond the level of most known confounders already accounted for, to fully explain the observed association.

Several limitations warrant acknowledgment. First, as with all electronic health record -based studies, the ascertainment of varicose veins depends on clinical documentation rather than systematic screening, and outcome underascertainment remains possible, particularly among patients with lower healthcare engagement or those managed in non-participating facilities. The slightly lower healthcare encounter rate in the VDD cohort may have reduced opportunities for varicose vein detection in this group. If present, such under-ascertainment would likely bias the association toward the null, suggesting that the observed HR may be conservative. Although the sensitivity analysis restricted to ambulatory encounters yielded consistent results, differential surveillance between cohorts cannot be fully excluded. Second, vitamin D status was defined using threshold-based categories rather than continuous measurements, which may introduce exposure misclassification and precludes examination of associations across the full spectrum of circulating 25(OH)D concentrations. Additionally, seasonal variation in 25(OH)D levels could not be standardized, although the requirement for two qualifying measurements and exclusion of individuals with discordant values within three years may mitigate the impact of transient seasonal fluctuations on exposure classification. Similarly, the TriNetX database does not capture detailed clinical severity information such as Clinical-Etiological-Anatomical-Pathophysiological (CEAP) classification, duplex ultrasound findings, or venous anatomical characteristics, although the secondary outcome analyses of varicose veins with ulceration, inflammation, and other complications provide a partial assessment of disease severity. Third, despite the rigorous propensity score matching framework, residual confounding from unmeasured variables cannot be entirely eliminated; in particular, physical activity level, occupational prolonged standing, sunlight exposure, socioeconomic status, dietary patterns, parity, and genetic predisposition to venous insufficiency were not captured in the TriNetX database and may have influenced the observed associations. Notably, inherited thrombophilia, including Factor V Leiden and prothrombin G20210A mutations, could not be reliably assessed because genetic thrombophilia testing is not routinely performed and is documented in only a small, non-representative subset of patients within the database; although anticoagulant use was included as a matching variable, residual confounding from undiagnosed thrombophilia cannot be excluded. Fourth, the causal directionality of the observed association cannot be established from observational data alone, and reverse causation, whereby subclinical venous disease reduces physical activity and sun exposure, cannot be completely excluded. Fifth, serum magnesium levels were not available for analysis; given that magnesium is a critical cofactor for vitamin D activation, unmeasured magnesium deficiency may have contributed to both the observed VDD and the associated varicose vein risk, and its potential confounding or mediating effect could not be assessed. Finally, as TriNetX predominantly comprises healthcare organizations in the United States, the generalizability of these findings to populations with different demographic profiles, dietary habits, or healthcare systems warrants caution.

## 5 Conclusion

In this large propensity score-matched cohort study, VDD was associated with an increased risk of incident varicose veins, including subtypes with ulceration and inflammation. The association was also observed among older adults and individuals with diabetes, suggesting that these groups may warrant particular attention in future research. These findings support the potential relevance of vitamin D status in the clinical evaluation of patients at risk for venous disease. However, given the observational design, the results should be interpreted as associations rather than evidence of causality. Whether correction of VDD can reduce the risk of varicose vein development remains uncertain and should be evaluated in prospective interventional studies.

## Data Availability

The datasets presented in this article are not readily available because the data was obtained from the TriNetX Global Collaborative Network. Data access can be obtained through a direct agreement with TriNetX. Requests to access the datasets should be directed to https://trinetx.com.
