# Supplementary material for: Association between vitamin D deficiency and incident varicose veins: a propensity score-matched cohort study
Source: Front Nutr. 2026 Jun 25;13:1870207. doi: 10.3389/fnut.2026.1870207 (PMC13345816; doi:10.3389/fnut.2026.1870207)
Supplement: Supplementary file 1 [file Table_1.DOCX]

**Supplemental Table 1. Codes Used for Cohort Definition, Inclusion/Exclusion Criteria, Outcome Definitions, and Variables for Propensity Score Matching**

| Domain | Variable | Codes / Definition |
| --- | --- | --- |
| Inclusion | Age | ≥40 years |
|  | Vitamin D measurement | LOINC/TriNetX: LG25965-1, 9034 (serum 25[OH]D) |
|  | VDD cohort | ≤19.9 ng/mL (two measurements) |
|  | Control cohort | ≥30.0 ng/mL (two measurements) |
| Exposure stability | VDD exclusion | Any 25(OH)D ≥30 ng/mL within 3 years |
|  | Control exclusion | Any 25(OH)D ≤19.9 ng/mL within 3 years |
| Outcome (primary) | Varicose vein | ICD-10-CM: I83 |
| Outcome (secondary) | Varicose vein subtypes | I83.0, I83.1, I83.8 |
| Validation outcomes | Osteoporotic fracture (positive control) | ICD-10-CM: M80 |
|  | Appendiceal disease (negative control) | ICD-10-CM: K38 |
|  | Healthcare utilization | Visit (TriNetX Visit) |
| Matching variables | Demographics | Age, sex, race |
|  | Comorbidities | E66 (obesity), E08–E13 (diabetes), I10 (hypertension), E78 (hyperlipidemia), N18 (CKD), F17 (nicotine), F10 (alcohol), J44 (COPD), I20–I25 (IHD), C00–D49 (cancer) |
|  | Others | K21 (GERD), D50 (IDA), E50–E64 (nutritional deficiency), E00–E07 (thyroid), I60–I69 (CVA), M30–M36 (CTD) |
|  | Medications | Vitamin D (VT500), steroids (H02AB), insulin (A10A), contraceptives (HS200), estrogen (HS300), anticoagulants (BL110), antiplatelets (BL117) |
|  | Laboratory | BMI (9083), albumin (9045), hemoglobin (9014), HbA1c (9037), eGFR (62238-1) |
| Major exclusions | Prior varicose vein | I83 |
|  | Other venous diseases | I86, I87, I80, I82 |
|  | Varices | I85 |
|  | Pregnancy-related venous disease | O22, O87 |
|  | Cardiopulmonary | I27, I50 |
|  | Liver disease | K70, K72 |
|  | Lymphedema | I89.0 |
|  | Thrombophilia | D68.5, D68.6 |
|  | Severe systemic disease | N18.4–N18.6, Z99.2 |
|  | Malabsorption | K50, K90.0 |
|  | Bariatric status | Z98.84 |
| Procedure exclusions | Venous intervention | CPT: 36468, 1006677, 1006680 |

**Supplemental Figure 1.** Flow diagram of cohort selection. The diagram illustrates identification of eligible adults aged ≥40 years with at least two serum 25-hydroxyvitamin D measurements in the TriNetX Global Collaborative Network, application of exposure definitions, inclusion and exclusion criteria, 1:1 propensity score matching, and the final analytic population. VDD, vitamin D deficiency.

**
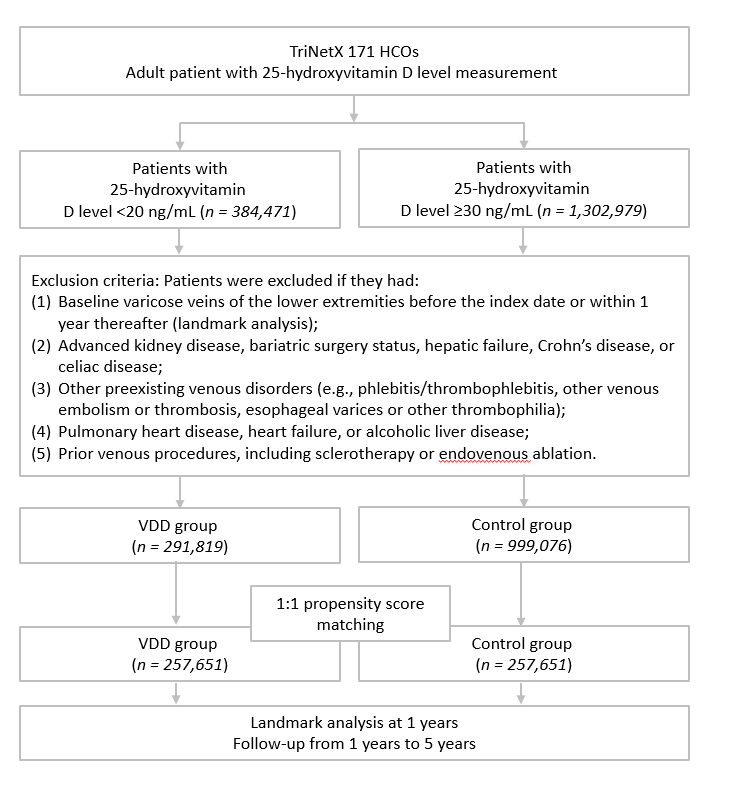
**
